# Future Aspects for Cannabinoids in Breast Cancer Therapy

**DOI:** 10.3390/ijms20071673

**Published:** 2019-04-03

**Authors:** Terézia Kisková, Felicitas Mungenast, Mária Suváková, Walter Jäger, Theresia Thalhammer

**Affiliations:** 1 Institute of Biology and Ecology, Faculty of Sciences, University of Pavol Jozef Šafárik in Košice, Šrobárova 2, 04154 Košice, Slovakia; 2 Department of Pathophysiology and Allergy Research, Center for Pathophysiology, Infectiology and Immunology, Medical University of Vienna, Währinger Gürtel 18-20, 1090 Vienna, Austria; theresia.thalhammer@meduniwien.ac.at; 3 Institute of Chemistry, Faculty of Sciences, University of Pavol Jozef Šafárik in Košice, Šrobárova 2, 04154 Košice, Slovakia; maria.suvakova@student.upjs.sk; 4 Department of Clinical Pharmacy and Diagnostics, University of Vienna, Althanstrasse 14, 1090 Vienna, Austria; walter.jaeger@univie.ac.at

**Keywords:** breast cancer, *Cannabis sativa*, cannabinoid receptor, cannabidiol, CBD, delta-9-tetrahydrocannabinol, THC

## Abstract

Cannabinoids (CBs) from *Cannabis sativa* provide relief for tumor-associated symptoms (including nausea, anorexia, and neuropathic pain) in the palliative treatment of cancer patients. Additionally, they may decelerate tumor progression in breast cancer patients. Indeed, the psychoactive delta-9-tetrahydrocannabinol (THC), non-psychoactive cannabidiol (CBD) and other CBs inhibited disease progression in breast cancer models. The effects of CBs on signaling pathways in cancer cells are conferred via G-protein coupled CB-receptors (CB-Rs), CB1-R and CB2-R, but also via other receptors, and in a receptor-independent way. THC is a partial agonist for CB1-R and CB2-R; CBD is an inverse agonist for both. In breast cancer, CB1-R expression is moderate, but CB2-R expression is high, which is related to tumor aggressiveness. CBs block cell cycle progression and cell growth and induce cancer cell apoptosis by inhibiting constitutive active pro-oncogenic signaling pathways, such as the extracellular-signal-regulated kinase pathway. They reduce angiogenesis and tumor metastasis in animal breast cancer models. CBs are not only active against estrogen receptor-positive, but also against estrogen-resistant breast cancer cells. In human epidermal growth factor receptor 2-positive and triple-negative breast cancer cells, blocking protein kinase B- and cyclooxygenase-2 signaling via CB2-R prevents tumor progression and metastasis. Furthermore, selective estrogen receptor modulators (SERMs), including tamoxifen, bind to CB-Rs; this process may contribute to the growth inhibitory effect of SERMs in cancer cells lacking the estrogen receptor. In summary, CBs are already administered to breast cancer patients at advanced stages of the disease, but they might also be effective at earlier stages to decelerate tumor progression.

## 1. Introduction: *Cannabis sativa* and Cannabinoids

*Cannabis sativa* (*C. sativa*) was known among ancient Asian, African, and European agricultural societies. Due to its hallucinogenic effects, *Cannabis sativa* was applied in religious ceremonies, but it was also widely used in fiber manufacturing, nutrition and medicine. However, in the early part of the last century, *C. sativa* lost its importance in industry and medicine [1,2]. At present, application of *C. sativa* in industry and medicine is experiencing a revival. Since 1990, *C. sativa* became important as a source of compounds to treat cancer and life-threating diseases. The *C. sativa* plant contains >500 chemical and biologically active compounds [3]. So far, 60 structures have been identified as belonging to the family of cannabinoids (CBs). CBs share a lipid structure featuring alkylresorcinol and monoterpene moieties (terpenophenols) [2,4].

**Figure 1:**
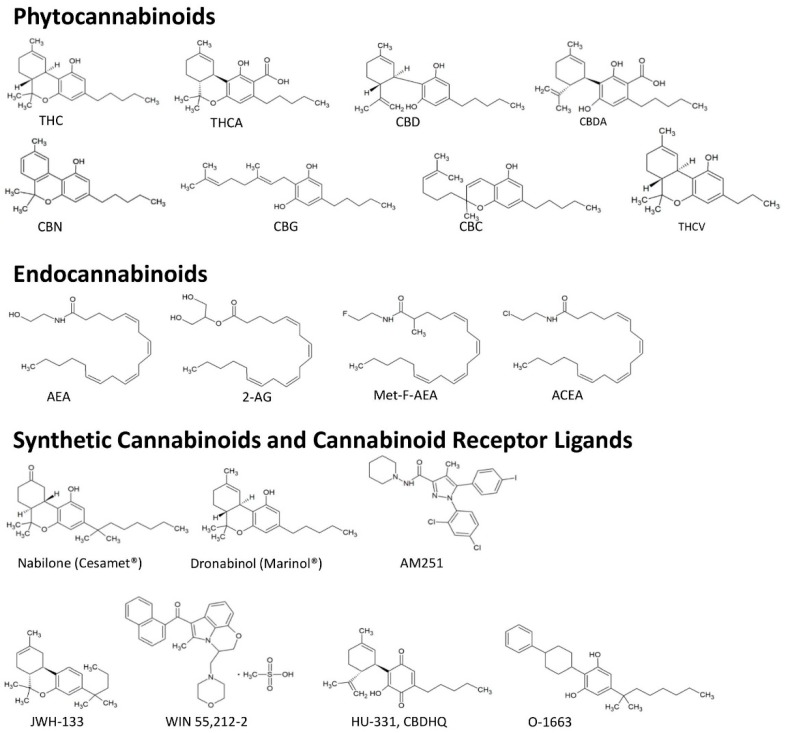
Chemical structures of cannabinoids. **Phytocannabinoids**—THC: Delta-9-tetrahydrocannabinol; THCA: Delta-9-tetrahydrocannabinolic acid; CBD: Cannabidiol; CBDA: Cannabidiolic acid; CBN: Cannabinol; CBG: Cannabigerol; CBC: Cannabichromene THCV: Tetrahydrocannabivarin. **Endocannabinoids**—AEA: Anandamide; 2-AG: 2-Arachidonoylglycerol; Met-F-AEA: 2-methyl-2’-F-anandamide; ACEA: Arachidonyl-2’-chloroethylamide. **Synthetic cannabinoids**—AM251: *N*-(Piperidin-1-yl)-5-(4-iodophenyl)-1-(2,4-dichlorophenyl)-4-methyl-1*H*-pyrazole-3-carbox amide; JW133: (6aR,10aR)-3-(1,1-Dimethylbutyl)-6a,7,10,10a-tetrahydro-6,6,9-trimethyl-6H-dibenzo[b,d]pyran-d5; WIN55,212-2: (*R*)-(+)-[2,3-Dihydro-5-methyl-3-(4-morpholinylmethyl)pyrrolo[1,2,3-*de*]-1,4-benzoxazin-6-yl]-1-naphthalenylmethanone mesylate; HU-331,CBDHQ: 3-Hydroxy-2-[(1*R*)-6-isopropenyl-3-methyl-cyclohex-2-en-1-yl]-5-pentyl-1,4-benzoquinone; O-1663: 5-(1,1-Dimethylheptyl)-2-(4-phenylcyclohexyl)-1,3-benzenediol.

Two CBs have been intensively investigated for their pharmacological properties: delta-9-tetrahydrocannabinol (THC) and cannabidiol (CBD); THC, but not CBD, exerts potent psychotropic effects (Figure 1). A high THC/CBD ratio is responsible for the euphoric, relaxing, and anxiolytic effects of medical cannabis (marijuana), whereas, a high CBD/THC ratio has a rather sedating effect [5].

Cultivation from different varieties of *C. sativa* produces two main varieties with distinct concentrations of CBs, and the discrimination from the THC/CBD ratio divides commercial cannabis strains into three principal chemotypes. Chemotype I flowers have the highest THC content (18–23%). Industrial *C. sativa* flowers (chemotype II and III flowers) contain less than 0.3% THC and CBD levels are 10–12% when calculated for dry weight [6]. Since there are still systematic differences in reports on the CB content and the relative stability of CB levels from different laboratories, a better standardization of CB analysis is urgently required.

Based on the ability of CBs to inhibit inflammation and block cancer cell proliferation, plant-derived and synthetic CBs have been investigated for their applications as antitumor drugs. Indeed, a growing number of reports on the role of receptors for CBs in tumor cells suggest that CBs with different properties that can block or activate CB-receptors (CB-Rs) may be useful in cancer treatment [7,8].

## 2. Mechanism of Cannabinoid Action

The term ‘endocannabinoid’ was invented in the mid-1990s after the discovery of membrane receptors for THC and their endogenous ligands. It now comprises a whole signaling system consisting of the ‘classical’ CB-Rs, their endogenous ligands, which are lipid signaling molecules called endocannabinoids, and the associated biochemical machinery, including precursor molecules, enzymes for synthesis and degradation, and transporter proteins, such as fatty acid binding protein and heat shock protein 70 [9]. There is now a growing number of endocannabinoid molecules known, which share a similar structure and are natural ligands of the two CB-Rs, CB1-R and CB2-R. They seem to be involved in an increasing number of pathological conditions. Plant-derived CBs (phytocannabinoids, phyto-CBs) as well as synthetic CBs interfere with the endocannabinoid system, and a number of pharmacological effects of phyto-CBs can be explained by this interference [10].

The most studied compounds of the endocannabinoid system are anandamide (N-arachidonoylethanolamine; AEA) and 2-arachidonoylglycerol (2-AG) (Figure 1). Each can activate both CB-Rs and both are synthesized on demand in response to elevations of intracellular calcium [11]. The biosynthesis of AEA, which was the first endocannabinoid identified, starts from the activation of N-acyltransferase (NAT), which transfers an acyl group to the membrane phospholipid phosphatidylethanolamine. In this way, N-acyl-phosphatidylethanolamine (NAPE) is generated. The NAPE-specific phospholipase D forms AEA from NAPE. The major biosynthetic pathway for 2-AG involves the sequential hydrolyses of inositol phospholipids via diacylglycerol (DAG) by phospholipase C and DAG lipase.

AEA and 2-AG are produced on demand by cells and work to maintain homeostasis [9]. They have a short half-life and are quickly degraded through transport protein reuptake and hydroxylation by either fatty acid amide hydrolase (FAAH) for AEA or monoacylglycerol lipase (MAGL) for 2-AG. Finally, arachidonic acid (AA) and ethanolamine, from AEA, and AA and glycerol, from 2-AG, are formed. Endocannabinoids are responsible for retrograde synaptic signaling in the central nervous system. They move across the synaptic cleft in order to bind and activate the presynaptic CB1-R, causing an inhibition of neurotransmitter release.

These compounds serve as a new class of endogenous signaling molecules involved in a plethora of physiological functions related to behavior, memory, temper, addiction, and reward systems, as well as cellular metabolism and energy regulation. Their synthesis occurs ‘on demand’ (no storage) with a very short half-life. Drugs influencing the endocannabinoid system (e.g., inhibitors of FAAH and MAGL) were developed to treat neurological diseases and neuropathic pain in cancer patients [12,13]. However, a tragic incidence at a phase I clinical trial with an FAAH inhibitor put its application into question [14].

Endocannabinoids work via specific G-protein coupled receptors (GPRs) CB-Rs (CB1-R and CB2-R). While AEA acts as a partial CB1-R agonist and is a weak CB2-R agonist, 2-AG is a strong CB1-R agonist. CB1-R and CB2-R belong to the seven-transmembrane-spanning receptor superfamily. The distinct tissue distribution of CB1-R and CB2-R allows a selective and cell-specific effect of receptor activation. CB1-R is highly expressed in brain areas related to cognitive functions, memory, anxiety, pain, sensory and visceral perception, motor coordination, and endocrine functions. Low expression levels are observed in the peripheral nervous system, testicles, heart, small intestine, prostate, uterus, bone marrow and vascular endothelium. CB1-R activations inhibit forskolin-stimulated adenylyl cyclase through activation of a pertussis toxin-sensitive G-protein, to inhibit N-, P-, and Q-type calcium channels, and activate inwardly rectifying potassium channels.

CB2-R is present at high levels in cells of the immune system. In glial cells, the spleen and tonsils, CB1-R levels are low. CB2-Rs are also present at a lower level in the heart, endothelium, bones, liver, and pancreas. Furthermore, a functionally relevant expression of CB2-Rs was also found in the brain [15]. Intracellular CB2-R dependent signaling pathways include Gi/o-dependent inhibition of adenylyl cyclase, stimulation of mitogen-activated protein kinase (MAPK), phosphoinositide 3-kinase (PI3K) and cyclooxygenase-2 (COX-2) signaling, and activation of de novo ceramide synthesis. Both CB-R types are highly expressed in a variety of cancerous tissues, and it is well established that CB2-R plays a crucial role in carcinogenesis and cancer progression. Therefore, CB2-R is now emerging as target for cancer treatment, although the exact role of CB2-R in cancer progression is still not completely elucidated [16].

**Figure 2:**
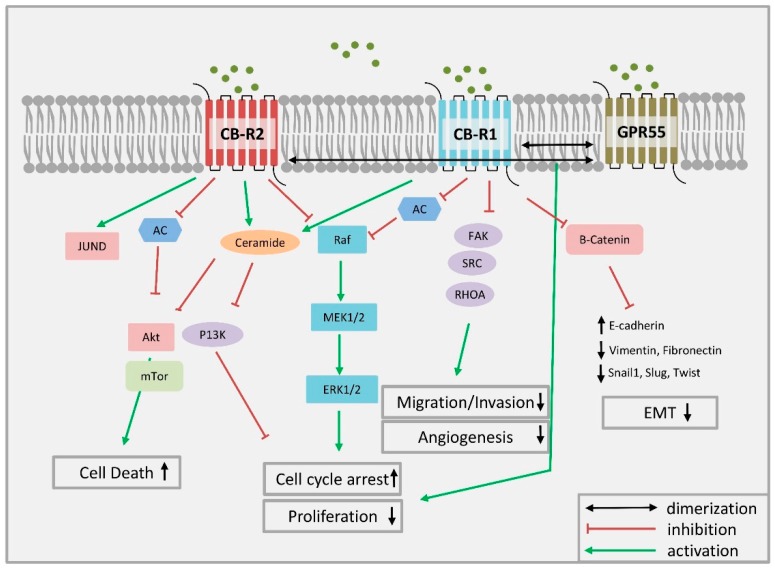
Mechanism of CB-R-mediated antitumor activity in breast cancer cells. By binding to CB1-R and CB2-R, CBs inhibit breast cancer cell proliferation through various mechanisms. They block cell cycle progression at the G1/S phase via CB1-R and at the G2/M phase via CB2-R activation. They induce breast cancer cell death via apoptosis, mediated by the activation of the transcription factor jun-D. In HER2-overexpressing breast cancer cells, they block cancer cell proliferation in culture and tumors by inhibiting Akt and ERK signaling. They also inhibit cell migration and angiogenesis via CB2-R. CB1-R activation inhibits the FAK/SRC/RhoA pathway leading to inhibition of cell migration. Cell migration blockade is also achieved by CB2-R activation through the inhibition of COX-2 and ERK signaling, which is important for triple-negative breast cancer. AC: adenylate cyclase; Akt: protein kinase B; CB-R: cannabinoid receptor; COX-2: cyclooxygenase-2; EMT: epithelial-mesenchymal transition; ERK: extracellular-signal-regulated kinase; FAK: focal adhesion kinase; GPR: G-protein coupled receptor; HER2: human epidermal growth factor receptor 2: MAPK: mitogen-activated protein kinase; mTOR: mammalian target of rapamycin; PI3K: Phosphoinositol-3-kinase; Raf: serine/threonine-protein kinase; RhoA: transforming protein RhoA; SRC: proto-oncogene tyrosine-protein kinase Src.

At molecular levels, the activation of CB-Rs confers signals of endo, phyto, and synthetic CBs (Figure 1) via inhibition or activation of a variety of signaling pathways [17] (Figure 2). An important signal transduction pathway regulated by CB-R is linked to the synthesis of ceramide with palmitoyl-transferase as the rate-limiting enzyme in ceramide synthesis [18]. Long-term treatment with ceramide, which activates the proto-oncogene serine/threonine-protein kinase (RAF1), leads to sustained activation of p42/p44 MAPK and induction of apoptosis, as demonstrated in a glioma cell line. This activation could be blocked by CB-R agonists, including THC, by the synthetic CB WIN55,212-2, and the endocannabinoids AEA and 2-AG. However, the duration of the activation of p42/p44 MAPK seems to be critical to the apoptotic response because a protective role of CBs against ceramide-induced apoptosis was also reported [19].

Importantly, CBs also bind and activate several other receptors, including the GPRs, GPR18, GPR55, and GPR119. Of particular interest is GPR55, which is activated by lysophospholipid and also by the endocannabinoids AEA and 2-AG. Downstream targets of GPR55 include phospholipase C (PLC), transforming protein RhoA (RhoA), Rho-associated protein kinase (Rock), extracellular-signal-regulated kinase (ERK), and p38 MAPK [20]. CB-Rs form heterodimers with other GPRs, e.g., GPR55, which consequently affects the functions of both receptors. Other GPRs, which are activated by CBs, are acetylcholine receptors and 2-alpha adrenoreceptors as well as opioid-, adenosine-, 5-hydroxytryptophan-, angiotensin-, prostanoid-, dopamine-, melatonin-, and tachykinin receptors. Furthermore, the peroxisome proliferator-activated receptors (PPARs) α and γ are also considered to be receptors for endocannabinoids [21].

## 3. Cannabinoids from *Cannabis sativa*

### 3.1. Cannabidiol (CBD)

CBD and its precursor cannabidiolic acid (CBDA) are the main phyto-CBs in industrial used *C. sativa* [3,22]. CBD works as an allosteric negative modulator of CB1-R and CB2-R activity [23,24]. Some of its pharmacological effects are caused by it binding to other GPRs and other receptors (see previous chapter). For example, the anticonvulsant, antispasmodic, anxiolytic, antiemetic, and neuroprotective effects of CBD are thought to be conferred by several GPRs in neuronal cells. CBD acts as a partial agonist for GPR18 and GPR55 and antagonizes the effects of THC [24].

The pharmacokinetic properties of all CBs are highly dependent on the route of administration. A high intra and intersubjective variability is common in humans. Extensive studies in animals, including rodents and dogs, indicated that a high amount of administered CBD is excreted unchanged or in its glucuronidated form. The most abundant metabolites are the hydroxylated 7-carbonyl CBD derivatives, which are excreted into urine either in their unconjugated form or as glucuronides. Lipid soluble CBs and their metabolites, found in blood and urine, can be stored in fat cells for up to several weeks. Typically, CB and its metabolites appears in the urine within 60 min with high concentrations for ≤4 h [25].

The 7-carbonyl metabolites confer anti-inflammatory properties in mice. In vitro studies revealed that they reduce nitric oxide (NO) formation and prevent the formation of reactive oxygen species (ROS). They also block the production of tumor necrosis factor (TNF)-α and other pro-inflammatory cytokines and transcription factors [e.g., interleukin (IL)-1β, IL-2, IL-6, IL-8 and nuclear factor (NF)-κB], and their effects are comparable to that of CBD. CBD is known to inhibit the metabolism of AA to leukotriene B4 via 5-lipoxygenase as part of its anti-inflammatory effect [26]. Although CBD was found to reduce the formation of ROS and NO in various cell lines and animal models of inflammation, there are also reports showing that CBD can induce ROS formation in cancer cells, leading to cytotoxicity [27].

### 3.2. Delta-9-tetrahydrocannabinol (THC)

THC is the main psychotropic constituent of *C. sativa* and is a CB1-R and CB2-R partial agonist. Thereby, the CB-R expression level and signaling efficiency of CB-Rs together with the release of endogenous CBs will influence its effects. Euphoria is among the most often observed psychotropic effects, but dysphoric reactions, including anxiety and panic reactions, as well as paranoia, are known. The absorption kinetics of THC (similar to those of other CBs) depend on the exposure route. Inhaled THC is rapidly distributed in the bloodstream, with peak levels observed at 2–10 min. Concentrations decline rapidly within 30 min and the formation of the psychoactive 11-hydroxy metabolite stops. After oral consumption, THC reaches peak levels after 2–4 h and the half-life of THC is 20–30 h. The oral bioavailability of the highly lipophilic THC and of other CBs is low and variable (6–20%). The hepatic cytochrome p450 system primarily metabolizes THC to many hydroxylated metabolites, which are mostly inactive [13]. However, the main active metabolite of THC is 11-hydroxy-delta-9-tetrahydFrocannabinol (11-OH-THC) with potent psychoactive activity. This metabolite is further degraded to mostly inactive metabolites, including 11-nor-delta-9-tetrahydrocannabinol-carboxylic acid, which is detectable in urine. The excretion of the metabolites through feces and urine lasts from hours to days, with a more prolonged elimination after chronicity of use. The presence of the metabolite in the urine indicates exposure to THC within the last 3 days.

The acute toxicity of CBs is low in adults, but toxic effects occur mostly through THC. Inhaled doses of 2–3 mg THC and ingested doses of 5–20 mg THC can lead to impaired attention and memory, as well as in executive functioning, and conjunctivitis is a common symptom. Higher doses in adults and oral 5–300 mg in pediatric patients can cause more severe symptoms such as hypotension, panic, anxiety, delirium, respiratory depression and ataxia. Furthermore, chronic application of THC may lead to attention and memory deficits, as well as loss of the ability to process complex information. In children, neurological abnormalities, including lethargy and hyperkinesis, can be signs of severe toxicity. As THC crosses the placenta and accumulates to significant concentrations in breast milk, THC consumption by pregnant and breast-feeding women may harm unborn and newborn babies [28].

Physiological effects of THC primarily take place in the central nervous system. Activation of CB1-R by THC leads to a disturbance in the gamma aminobutyric acid/glutamatergic neurotransmission system and the release of dopamine [24]. Thereby, the expression level and signaling efficiency of CB1-R determines the psychotropic effects of THC.

As described for CBD, antiproliferative actions of THC in tumor cells are caused by the activation of CB-Rs, which influence various signaling mechanisms (Figure 2). Activation of CB2-R impairs cell cycle progression by downregulating cell division control 2 (Cdc2) and inducing cell cycle arrest at the G2/M phase. Furthermore, CB2-R causes an activation of a member of the activating protein 1 transcription family, transcription factor jun-D, preventing cell proliferation and inducing apoptosis [29,30].

CB2-R activation also induces PPARγ-regulated pathways in carcinoma cells. In this way, CBs promote the expression of intercellular adhesion molecule 1. This process results in an enhancement of cancer cell adhesion to lymphokine-activated killer cells and causes cancer cell lysis.

THC was shown to antagonize the tumor-promoting GPR55, both at the single receptor level and within the CB2-R-GPR55 heterodimers. These heterodimers of CB2-R and GPR55 influence tumor growth by modulating cyclic adenosine monophosphate (cAMP) signaling and the ERK-1/2 pathways [31,32].

### 3.3. Minor Phytocannabinoids

Other CBs from *C. sativa* were also found to have anti-inflammatory and analgetic effects. Some of these CBs were found to improve the effects of inflammatory diseases in the gut and stimulate bone formation. These effects are conferred by the activation of CB-R and other receptors. Their concentration varies between different *C. sativa* strains but is generally as low as 2%. However, the concentrations of these CBs may reach significant levels in special cultivated strains. Additive or synergistic interactions between CBD, THC with minor phyto-CBs, or non-CBs, such as terpenes, in the extracts may increase the therapeutic efficiency of the extract for the treatment of inflammation and cancer [12,33].

#### Other CBs from *C. sativa*

Cannabinol (CBN) is a non-psychoactive CB with a higher concentration in aged plants, or in degraded or oxidized CB preparations. Pharmacologically relevant quantities are formed as a metabolite of THC. CBN is a partial CB1-R agonist, but it has a higher affinity to CB2-R than to CB1-R.

Cannabigerol (CBG) was found to improve digestive functions and has powerful antiemetic and anti-inflammatory effects. CBG is a partial agonist for CB1-R and CB2-R [34]. It may be used for the treatment of neurological disorders.

Cannabichromene (CBC) has mild psychotropic effects and may stimulate bone growth [35]. It also has anticonvulsive effects. It is may be used in the treatment of hypomotility, catalepsy and hypothermia.

Tetrahydrocannabivarin (THCV) works as a potent CB-R partial agonist in vitro. THCV interacts with CB1-R when administered in vivo, behaving as a CB1-R antagonist at low doses and as an agonist at higher doses [24]. THCV has antibacterial and antiviral properties and is also thought to prevent obesity. It may additionally have some anti-convulsive properties [36].

### 3.4. Drugs Based on CBs from C. sativa

Nabilone (Cesamet^®^) and Dronabinol (Marinol^®^) are synthetic molecules that mimic the pharmacological activity of THC. Their chemical structures are presented in Figure 1.

Nabiximol (Sativex^®^) was first approved as a botanical drug in the UK in 2010. The aerosol mouth spray contains an extract from the *C. sativa* plant and flowers derived from two cannabis plant varieties. It contains nearly equal amounts of THC and CBD, but also minor quantities of CBs, flavonoids and terpenes from the plant.

### 3.5. Synthetic Cannabinoid Analogues

To target CB-R mediated pathways, compounds with different chemical structures were screened for CB-R receptor ligand activity. A number of these compounds were investigated in cell culture and animal tumor models to determine their antineoplastic effects. For relevant reviews, see references [37,38,39,40,41]. Their chemical structures are depicted in Figure 1 and their effects are discussed in the following chapters.

## 4. Cannabinoids in Breast Cancer

### 4.1. Molecular Effects of CBs in Breast Cancer

Breast cancer is the most frequently diagnosed cancer in women worldwide. There is also an increasing tendency for aggressive subtypes of breast cancer, particularly in women of younger ages [6,42]. Although the main intrinsic molecular subtypes are breast cancer hormone receptor-positive, human epidermal growth factor receptor 2 (HER2)-negative luminal A and B tumors, HER2-enriched tumors and triple-negative tumors, which are usually the most aggressive type, have been identified. As these molecular subtypes differ in the course of the disease and the clinical outcome, individualized therapies will achieve a better outcome for individual patients [43]. Interestingly, data from preclinical in vitro and in vivo studies identified various antitumor activities of plant-derived and synthetic CBs, although there are some studies in which CBs might also promote tumor progression. The relevant data are summarized in the following chapters and the kinetic data for individual CBs are summarized in Table 1.

### 4.2. Cannabinoid Receptor Signaling

Breast cancer cell lines express CB2-R at high levels but levels of CB1-R are rather low [44]. On a microarray performed on human breast cancer samples with different histological features, CB1-R immunoreactivity was found in 28% of carcinomas and CB2-R was identified in 72% of carcinomas. No significant CB1-R and CB2-R immunoreactivity was detected in non-transformed mammary tissue [45]. CB2-R expression in breast cancer correlates with the aggressiveness of the tumors. Estrogen- and/or progesterone receptor-negative tumors, which are more aggressive than tumors expressing steroid-hormone receptors, express higher levels of CB2-R, which usually have a better prognosis [46]. In particularly difficult to treat triple-negative tumors (lacking the expression of receptors for steroid hormones and *HER2*/neu (human epidermal growth factor receptor 2/*erb*-B2, and tumors expressing HER2/*erb*-B2 but no steroid hormone receptors, increased CB2-R levels are nearly always observed. These tumors are usually poorly differentiated, contain highly proliferative and invasive growing cells, and have a higher probability for early local tumor recurrence and formation of distant metastases. Therefore, they usually have a poorer prognosis than steroid hormone receptor positive tumors [47,48]. To treat these tumor entities, targeting CB-associated pathways could be a promising treatment option and might also work in patients suffering from a relapse with an anti-HER2 targeted therapy.

In addition to CB2-R and CB1-R, alternative CB-Rs are also of interest for breast cancer therapy. High expression levels of GPR55 were found in human breast tumors and were related to worse prognoses. GPR55 was also highly expressed in MDA-MB-231 cells, a human breast cancer cell line with considerable metastatic potential (compared with less-metastatic MCF-7 cells) [49]. The proliferative effects mediated by GPR55 are thought to be a result of ERK activation and downstream expression of proto-oncogene c-FOS [20].

### 4.3. The Effect of Cannabinoids in Breast Cancer Cell Lines

#### 4.3.1. Phytocannabinoids and Synthetic Analogues

In 2006, Ligresti et al. demonstrated that CBD caused a potent and selective inhibition of breast cancer cell growth [50]. A number of breast cancer cell lines, such as estrogen receptor (ER)-positive MCF-7, ZR-75-1, and T47D cells, and ER-negative cell lines MDA-MB-231, MDA-MB-468 and SK-BR3, are sensitive to the antiproliferative effects of CBD [27,50,51,52,53]. CBD interferes with cell cycle progression and causes an increase in the number of breast cancer cells in the resting G0 stage and in the G1 compartment. At higher concentrations, CBD causes cell death [46]. Shrivastava et al. showed that in CBD-treated breast cancer cells, a complex interplay between apoptosis and autophagy exists. In the MDA-MB-231 breast cancer cell line, CBD leads to an increase in the generation of ROS, which finally results in an induction of apoptosis and autophagy [27]. Using the MDA-MB-231 breast cancer cell line, it was further shown that beclin 1, a protein that interacts with either B cell lymphoma-2 or PI3K, plays a central role in the induction of autophagy and cell death. CBD causes apoptosis through the production of ROS by changing the mitochondrial permeability transition pore opening, as first demonstrated in human monocytes [54]. CBD inhibits protein kinase B (Akt) and mammalian target of rapamycin (mTOR) signaling and induces autophagy and cell death under oxidative stress conditions. An interplay among decreased mTOR and cyclin D1 together with an upregulated PPARγ expression promotes the induction of apoptosis, a process that is independent of the expression of ERs [55].

In both ER-positive and ER-negative breast cancer cells, CBD activates the intrinsic apoptotic pathway by changing the mitochondrial membrane potential, activating the translocation of the BH3 interacting-domain death agonist to the mitochondria, and releasing cytochrome C from mitochondria [27].

CBD also inhibits the invasiveness of aggressive MDA-MB-231 and MDA-MB-436 breast cancer cell lines by downregulating inhibitor of DNA binding 1 (ID-1), a transcriptional regulator, which stimulates the metastasis of breast cancer [51]. In a mouse model of advanced breast cancer with lung metastases, CBD reduced the degree of metastasis by downregulating ID-1. However, CBD caused only a moderate increase in survival in this model. The resorcinol derivative O-1663 was proposed as a selective for CB2-R, which prolonged the survival more efficiently than the parent compound in mice with advanced breast cancer. O-1663 inhibited ID-1, stimulated ROS production, and increased autophagy and apoptosis [56].

An important finding was that CBD improved the response to treatment with cytarabine and vincristine in cancer cells [57]. In vitro studies showed greater antitumor activity when combining CBs and radiotherapy. Survival of patients treated with CBs could be significantly increased by the incorporation of CBs in smart biomaterials for sustained delivery [58].

As CBD is derived from CBDA by decaboxylation, it was investigated whether CBDA is also biologically active [59]. Indeed, CBDA prevents migration of triple-negative MDA-MB-231 human breast cancer cells via CB2-R activation by modulating the expression and activity of COX-2 [59,60,61,62]. Furthermore, CBDA inhibits the growth and migration of breast cancer cells via the inhibition of cAMP-dependent protein kinase A via activation of the small GTPase, RhoA [61]. It also causes a downregulation of the enhancer of breast cancer metastasis ID-1 [62].

Resembling the effects of CBD, THC has pro-apoptotic effects in a number of breast cancer cell lines (EVSA-T, MDA-MB-231, MDA-MB-468, SKBR-3, MCF-7 and T-47D) [46]. It reduces cell cycle progression and induces apoptosis in hormone-sensitive and hormone-resistant human breast cancer cell lines. In this way, THC induces cell cycle arrest at the G2/M transition, causing downregulation of Cdc2 and inducing ROS formation to induce cancer cell death. This mechanism is also seen in a number of other cancer cell types e.g., glioma cells [46].

CBs are favorable for antitumor therapies, as they are potent inhibitors of the inflammatory process, primarily via CB2-R activation. As demonstrated in MCF-7 and MDA-MB-231 cells, THC suppressed the cell-mediated T helper (Th)1 response and enhanced Th2-associated cytokine secretion [63]. Furthermore, THC prevented activation of inflammatory signaling pathways, such as the NF-κB, MAPK, and JAK-signal transducer and activator of transcription (STAT) pathways in immune cells. Therefore, CBs may be a potent treatment option against breast cancer subtypes accompanied by strong inflammation, as well as against non-malignant inflammatory disorders [64].

Triple-negative breast tumor cells express basal markers, such as epidermal growth factor receptor (EGFR) and cytokeratin 5/6 at high levels. In these tumors, higher expression levels of basal markers, such as EGFR, are associated with a poorer outcome. Although EGFR inhibitors are effective in treating cancer, the early onset of drug resistance limits their therapeutic success [65]. In SUM159 and SCP2 human tumor cells, as model cells for triple-negative breast cancer, CBD effectively inhibited epidermal growth factor (EGF)-induced tumorigenic properties of these cancer cells by obstructing signaling pathways for EGFR, Akt, ERK, and NF-κB. Furthermore, CBD is able to block the secretion of matrix metalloproteinases (MMPs) and the effects of EGF on the cytoskeleton [66].

Studies in breast cancer cell lines and animal models showed that an extract from *C. sativa* was more potent than CBD and THC. Minor CBs in the extract may also contribute to the observed anticancer activity by modulating various targets in the pro-oncogenic pathways leading to an “entourage effect” against cancer cells [67].

#### 4.3.2. Endocannabinoids

Of the endocannabinoids, AEA was characterized for its antitumoral properties in vitro and in vivo. AEA modulates the cAMP/protein kinase A and MAPK kinase pathway to exert antiproliferative effects in breast cancer cells [68]. AEA inhibits the proliferation of breast cancer cells through nerve growth factor (NGF) and prolactin, by downregulating the NGF receptor and prolactin receptor, respectively [69]. The inhibitory effect of AEA on prolactin receptors may regulate the cancer-directed immune system, as prolactin is a potent endogenous proliferative agent of B and T cells [70]. Furthermore, AEA inhibits cell cycle progression by preventing G1/S transition, as demonstrated in the human prolactin sensitive breast epithelioid EFM-19 cell line [70]. Similar to AEA, the endocannabinoid 2-AG also exerts antiproliferative activity in MCF-7 and T-47D cells [69], as well as in EMG-19 breast cancer cells [70]. The synthetic AEA analogue Met-F-AEA has an increased binding affinity and selectivity for CB1-R compared with AEA, which leads to dose-dependent inhibition of cell proliferation in MDA-MB-231 cells [71]. This analog also inhibits the epithelial-mesenchymal transition of cancer cells, thereby preventing invasive growth and metastases of cancer cells [72], indicating that in addition to CB2-R, CB1-R is also a main target for the observed anticancer effect.

Other synthetic cannabinoid derivatives, e.g., ACEA, a selective CB1-R agonist, and AM251, a selective CB1-R antagonist, were investigated for their effects on breast cancer stem cells. While ACEA decreased the invasive potential, AM251 increased the invasive power of breast cancer stem cells, indicating that CB1-R contributes to the stem cell properties in breast cancer [73]. Furthermore, a number of other synthetic CBD analogues, such as O-1663 and HU-331, showed antiproliferative activity on breast cancer cells; these analogues were previously reviewed [38].

### 4.4. Preclinical Evidence of the Effects of CBs in Animal Models

It was demonstrated that CBD had favorable effects in a mouse model of cisplatin-induced nephropathy. Cisplatin induced the expression of superoxide-generating enzymes, enhanced the formation of ROS and inducible NO synthase, and promoted apoptosis. It reduced inflammation by inhibiting TNF-α and IL-1β in the kidneys of the mice, leading to an improved renal function [74]. At a dose of 5 mg/kg body weight, CBD inhibits breast tumor growth and reduces tumor volume in xenografts in athymic nude mice, leading to prolonged survival of the animals [50,53].

Important for HER2-positive tumors, an association between the levels of CB-Rs and HER2 in human breast cancer was identified in the study by Caffarell et al. (2010) [45]. In total, 91% of the CB2-R-positive tumors were also positive for HER2 [75]. The levels of CB2-R in breast cancer are strongly related to the aggressive growth of the tumor, as CB2-R activation triggers signaling pathways that drive the proliferation and survival of cancer cells, tumor angiogenesis and epithelial-mesenchymal transition, promoting tumor cell migration, and invasion. Among these pathways are the PI3K/Akt and the ERK/MAPK cascades [75].

For the consideration of CBs in the therapy of breast cancer patients with difficult to treat HER2 expression tumors, a combination of CBs with HER2-targeted therapies, such as the tyrosine kinase inhibitor lapatinib, may be promising, as previous studies showed that CBs enhance the antitumor effects of the drugs. Moreover, this effect was also shown for the application of CBs with standard chemotherapeutic drugs such as cisplatin [67].

In a clinically relevant mice model of HER2-posititive cancer (the polyoma middle T oncoprotein transgenic mice, MMTV-neu mouse) selective overexpression of HER2 in the mammary epithelium resulted in the formation of focal tumors in the breast and lung metastases. These tumors also expressed CB2-R. Long-term treatment of these mice with either THC or the synthetic JWH-133 delayed the onset and progression of the tumors [47]. JWH-133 is as effective as THC in reducing tumor progression, without the psychoactive effects of THC. This tumor preventive effect was attributed to the blocking of the PI3K/Akt/mTOR signaling pathway via downregulation of the Akt kinase. Furthermore, the formation of metastatic lesions in the lung was reduced by downregulation of the metalloproteinase MMP2, which degrades the extracellular matrix, and the upregulation of the metallopeptidase inhibitor tissue inhibitor of metalloproteinases 1. JWH-133 also reduced vascular endothelial growth factor (VEGF) secretion, preventing tumor angiogenesis [45]. An antineoplastic effect was also shown for Met-F-AEA as it blocked the activity of the p21 ras oncogene and reduced tumor angiogenesis and VEGF expression [76,77].

A recent study explained how THC can exert an antitumor effect in HER2 positive breast cancer cells; HER2 forms heterodimers with CB2-R and the expression of these heterodimers correlates with a poor patient prognosis. By binding to CB2-R, THC is able to disrupt these HER2-CB2-R complexes, which leads to the inactivation of HER2 and its degradation [78].

CBD inhibited the growth of triple negative breast tumors in a 4T1.2 mouse model, where the tumor volume and tumor weight was greatly reduced. Furthermore, reduced tumor vascularization, reduced expression of EGFR, as well as reduced phosphorylation of Akt and ERK, will prevent tumor progression [66]. The synthetic CB derivative WIN-55,212-2 caused breast cancer suppression through a coordinated regulation of the COX-2/prostaglandin E2 signaling cascade [44]. In this model, WIN55,212-2 administered in combination with doxorubicin, enhanced the anticancer effect of the standard chemotherapeutic drug. WIN55,212-2 induced cell cycle arrest and apoptosis and inhibited the proliferation, migration, and invasion of other cancer types, including prostate cancer [44]. Similarly, the synthetic CB analogue HU-331 was shown to inhibit tumor growth in nude mice xenografts without significant signs of toxicity in healthy organs [79].

Furthermore, CBD was also found to modulate the tumor environment [66]. CBD reduces the secretion of cytokines such as granulocyte-macrophage colony-stimulating factor from cancer cells. Consequently, a reduced recruitment of macrophages from the tumor microenvironment by the cancer cells will suppress the angiogenesis in the tumor [28]. This will limit the supply of nutrients and oxygen required for tumor growth [31].

### 4.5. Effect of Cannabinoids Related to Estrogen

Estrogens, particularly the most potent estrogen 17β-estradiol (E2), bind to ERα and ERβ to mediate the transcription of target genes, which regulate cell metabolism, cell growth, differentiation, and survival. Transcription starts after binding of the estrogen/ER complexes to estrogen response elements in DNA. Furthermore, a variety of non-genomic effects of estrogens are mediated by their influence on cellular signaling pathways for the regulation of growth and differentiation.

In addition to the classical ERs, a G-protein coupled receptor for estrogen (GPER, also known as GPR30) has been identified in the plasma membrane of a great variety of cells. It is a major mediator of estrogen’s rapid cellular effects [80]. So far, the role of GPER in breast cancer progression is still not fully elucidated. Nevertheless, it has been shown that in patients with ER-positive breast cancer treated with tamoxifen, the expression of GPER is negatively correlated with relapse-free survival. In these patients, GPER is considered to be an independent prognostic parameter for a poor outcome. In triple-negative breast cancer, GPER expression seems to be associated with a younger age and a more aggressive disease (reviewed in [81]). The progression of estrogen-related cancer is promoted by GPER activation through the MAPK, PI3K, and PLC signaling pathways [81]. These pathways are also affected by CB-R signaling and potential interaction is expected. A concise review on overlapping pathways between CB-Rs and estrogens was previously conducted [82].

Pharmacological targeting of ERα has been proved to be effective for the prevention and treatment of breast cancer [83]. The majority of newly diagnosed breast cancer (>70%) express ERα and ERβ and are sensitive to estrogen-mediated growth stimulation. Estrogen induces the expression of genes associated with cellular proliferation and survival and contributes to breast cancer development and progression. Importantly, estrogen-sensitive tumors are successfully treated by an antihormonal therapy, and the expression of ERα is a positive prognostic marker for the patient’s risk of a future outcome [43,84]. Also, high levels of ERβ are associated with a better prognosis for the survival [85]. Although CBs do not bind to ERs [86], THC was found to exert antiestrogenic activities in breast cancer cell lines. Both estrogen and CBs influence pathways associated with cell growth, cell death, and tumor progression, and their antagonistic effects on pathways involving adenylate cyclase, MAPK, ERK, PI3K, and J-Jun may maintain homeostasis between cell survival and cell death [82]. Thereby, the CB-R-induced activation of ERβ-mediated transcriptional activation will disrupt ERα signaling, leading to a reduced expression of estrogen-regulated genes that promote cell growth [87].

In women at reproductive ages, E2 is secreted by the ovaries and taken up by breast cancer cells [88]. However, in post-menopausal women with the highest rate of breast cancer, E2 is locally formed from inactive estrogen precursors, such as estrone sulfate via estrogen sulfatase and from androgens via aromatase. Estrone sulfate, which is the most prominent estrogen in post-menopausal women and may be formed from androgenic precursors, are taken up from circulation [88].

For the treatment and recurrence prevention of ER-positive breast cancer, a long-term treatment with the selective estrogen receptor modulator (SERM) tamoxifen is now standard. Tamoxifen effectively blocks estrogen-related growth of cancer cells and increases the disease-free and overall survival in patients with ER-positive breast cancer. It is still the therapy of choice for the treatment of ER-positive breast cancer in premenopausal women [65,78]. Studies involving cancer cell lines showed that tamoxifen, a hydroxylated, biologically active metabolite, and several other newer SERMs, act as inverse agonists for CB1-R and CB2-R with considerable affinity (between nM and low µM concentrations). In ER-lacking cancer cells, tamoxifen modulates adenylate cyclase activity and causes an increase in the intracellular cAMP by modulating CB-R activity [89]. In ER-negative breast cancer cell lines, tamoxifen also increases intracellular calcium levels via CB2-R activation [64].

**Figure 3:**
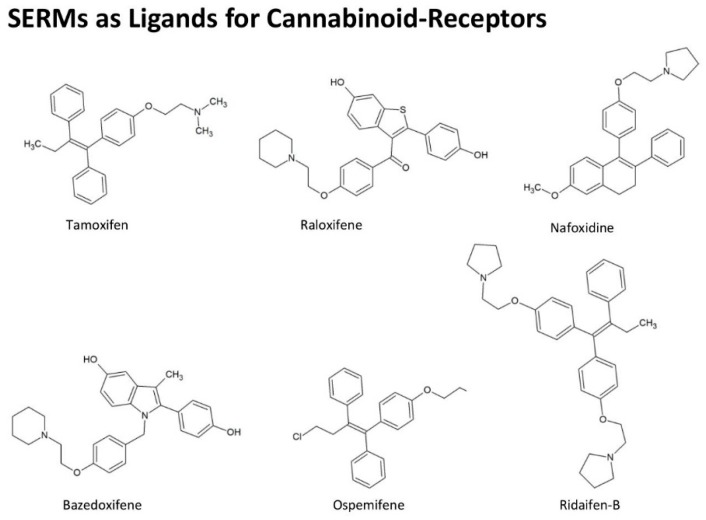
Chemical structures of SERMs. CBs: cannabinoids; SERMs: selective estrogen receptor modulators.

Additionally, several newer SERMs were identified as CB-R agonists. Chemical structures of selected SERMs are presented in Figure 3. Tamoxifen analog ridaifen-B from the group of ridaifen compounds was found to inhibit the growth of various cancer cell lines. However, they lack the affinity for ER binding. Ridaifen analogs are inverse agonists of CB2-R and have a potent anti-inflammatory effect. In lipopolisaccharide-activated macrophages, ridaifen analogs cause a reduction in the levels of NO and block pro-inflammatory cytokine secretion. The compounds also exhibit a pronounced anti-osteoporotic effect as they inhibit bone resorption by osteoclasts, preventing differentiation of bone marrow macrophages to osteoclasts, an effect which is partly due to CB2-R activation [90].

Further studies for new CBs with improved pharmacological properties compared with tamoxifen, lead to the identification of selective CB1-R and CB2-R modulators. An example of a selective CB1-R agonist is triphenylethylene ospemifene, whereas bazedoxifene, possessing an indole structure, binds to CB2-R [91]. The tetrahydronaphthalene nafoxidine and the benzothiophene raloxifene are effective in the endocrine treatment of post-menopausal breast cancer patients. These new SERMs reduce the basal G-protein activity and modulate the levels of intracellular cAMP, to block survival pathways for tumor cells [92].

As high CB2-R expression levels are found in ER-positive breast cancer, and the expression of CB2-R is negatively correlated with the prognosis for the tumor outcome, the development of novel SERMs that target ERα and CB2-R might be clinically relevant for future personalized cancer therapies [92]. In addition to their effects mediated by ERα and CB-R, SERMs block cancer cell growth through pathways independent of those receptors. For example, the SERM bazedoxifene is known to block the interaction between IL-6 and GP130 by activating STAT3 [93].

## 5. Current Therapeutic Application of Cannabinoids in Cancer Patients

At present, clinical trials on the effects of CBs from *C. sativa* in cancer patients are rare [55]. From the *C. sativa*-derived CBs, non-psychoactive CBD has been studied as an anticancer agent based on its in vitro and in vivo activity against tumor cells. On the other hand, THC was applied for its valuable effects in the palliative care of patients with advanced stages of cancer. However, not all molecular mechanisms through which CBs exert antitumoral activities are fully elucidated. With an increasing number of legal changes in the different countries that now allow patients to take CBs for the management of cancer-related symptoms, further studies may be conducted, which will improve the knowledge of the antitumoral effects of CBs.

Clinical studies (available at: https://www.cannabis-med.org/studies/study.php) monitoring the effects of CBs in patients with different late stages of cancer given a cannabinoid spray (Sativex^®^ containing THC and CBD at a ratio of 27:25 mg/mL) showed that this preparation is well tolerated and brought pain relief for ≤60% of patients suffering severe pain. The antiemetic, orexigenic, and anxiolytic effects of the CBs lead to an improved quality of life for cancer patients. Therefore, application of CBs in the palliative care of patients is well established. CBs are also successfully applied to treat muscle spasms and pain in patients with advanced multiple sclerosis, due to its analgesic and anticonvulsive effects. In these patients, dose-dependent adverse effects such as dizziness, gastrointestinal discomfort and confusion were reported [94].

Clinical studies showed that application of the synthetic CBs dronabinol and nabilone are only moderately effective for relieving cancer-related pain, but they improve chemotherapy-induced nausea and anorexia in most patients. Their antiemetic effect of synthetic CBs is superior to that of many neuroleptics. In particular, the ability of synthetic CBs to reduce delayed emesis after chemotherapy is comparable to that of serotonin receptor antagonists. Therefore, both CBs have been recommended for therapy-resistant nausea and vomiting caused by chemotherapy. Moreover, dronabinol proved to be effective in improving anorexia in patients with AIDS and may also benefit patients with an advanced stage of cancer for proper nutrition [95].

A study showed that a combination of CB drugs with opiates reduced chronic pain in ~27% of patients receiving oxycodone or morphine analgesics. No serious adverse effects were reported [96]. To reach a significant reduction in opioid dependence and achieve a reduction in the use of prescription medication for pain and cancer-related malaise, a concomitant application of CBs and opioids might be considered. Further clinical studies are required to introduce a wider application of CBs for substituting opioids at least partly in palliative therapy [1].

## 6. Summary

Many constituents of *C. sativa*, such as CBD and THC, exhibit beneficial anti-inflammatory or antitumoral properties. They act through the CB-Rs, CB1-R, and CB2-R. The latter receptor is highly expressed in cells of the immune system and both receptors are abundantly present in breast cancer cells. CB-R expression and activity determine the effects of CBs, but also of other drugs applied in the treatment of hormone-sensitive breast cancers. This was shown for the SERMs tamoxifen and raloxifene, which interfere with CB-R signaling. By influencing the tumor microenvironment and the immune system, blocking the expression of COX-2 and the proto-oncogene c-FOS and interfering with the EGF/EGFR pathway, they are able to reduce inflammation, inhibit tumor cell growth, induce apoptosis, and cause autophagy. This is important for HER2-positive tumors, where an increased CB2-R expression leads to activation of the HER2 pro-oncogenic signaling via the proto-oncogene tyrosine-protein kinase Src. On the other hand, CBs may enhance the proliferation of tumor cells by suppressing the immune system or by activating mitogenic factors.

Taken together, CBs are promising agents for inhibiting breast cancer progression. However, to develop safe therapeutic drugs, a further examination of the molecular pathways associated with CB activities is required.

## Figures and Tables

**Table 1:** Antitumoral activity of CBs in hormone-dependent and –independent breast cancer cell lines.

CB	Cell Line	IC_50_	Antitumoral Activity	Receptor Mechanism	Citation
**THC**	MDA-MB-231 MDA-MB-468 SKBR-3 MCF-7 EVSA-T T-47D	5.0 ± 1.2 µM 4.4 ± 0.3 µM 4.5 ± 0.4 µM 10.2 ± 0.7 µM 4.0 ± 0.1 µM 6.7 ± 0.2 µM	Induction of apoptosis Cell cycle arrest, Inhibition of G2-M transition via downregulation of Cdc2	CB2-R	[44]
	MDA-MB-231 MCF-7 4T1	n.d.	Increased production of IL-4 and IL-10 Suppression of the cell-mediated Th1 response and enhancement of the Th2-response	CB1-R CB2-R	[45]
	MDA-MB-231 MDA-MB-468	1.2 µmol/L 2.5 µmol/L	Antiproliferative activity Reduction of invasiveness via ID-1	n.d.	[46]
	MCF-7 MDA-MB-231	14.2 ± 2.1 µM 24.3 ± 4.2 µM	Inhibition of cell growth	CB2-R	[47]
**THCA**	MCF-7 MDA-MB-231	9.8 ± 0.4 µM 18.2 ± 5.3 µM	Inhibition of cell growth	CB2-R	[47]
**CBD**	MDA-MB-231 MCF-7 SK-BR-3 ZR-75-1 MCF-10A (n.m.)	n.d.	Inhibition of cell viability Induction of apoptosis/autophagy No influence on cell viability	CB1-R CB2-R TRPV	[27]
	MDA-MB-231 MCF-7	8.2 ± 0.3 µM 10.6 ± 1.8 µM	Inhibition of cell viability Cell cycle arrest at the G1/S transition Induction of apoptosis via pro-caspase-3 cleavage to caspase-3, induction of endoplasmic reticulum stress, inhibition of mTOR and Akt	CB2-R	[47]
	MDA-MB-231 T-47D	2.2 µM 5.0 µM	Induction of apoptosis, inhibition of mTOR, upregulation of PPARγ	n.d.	[48]
	MDA-MB-231 MDA-MB-436	1.3 µmol/L 1.6 µmol/L	Antiproliferative activity Invasiveness reduction via ID-1	n.d.	[46]
**CBDA**	MDA-MB-231	>100 μM	Inhibition of cell migration by modulating the activity and expression of COX-2	CB1-R CB2-R	[49,50,51,52]
	MDA-MB-231	25 μM	Inhibition of cAMP-dependent protein kinase A via activation of the small GTPase, RhoA	CB1-R CB2-R	[51]
	MDA-MB-231	>25 µM	Invasiveness reduction via ID-1 and SHARP1	n.d.	[52]
	MCF-7 MDA-MB-231	21.7 ± 3.2 µM >25 µM	Inhibition of cell growth	CB2-R	[47]
**CBN**	MDA-MB-231 MDA-MB-468	1.2 µmol/L 2.6 µmol/L	Antiproliferative activity Invasiveness reduction via ID-1	n.d.	[46]
**CBG**	MDA-MB-231 MDA-MB-468	2.3 µmol/L 2.1 µmol/L	Antiproliferative activity Invasiveness reduction via ID-1	n.d.	[46]
	MCF-7 MDA-MB-231	9.8 ± 3.4 µM 20.4 ± 2.6 µM	Inhibition of cell growth	CB2-R	[47]
**CBC**	MCF-7 MDA-MB-231	14.2 ± 1.4 µM >25 µM	Inhibition of cell growth	CB2-R	[47]
**AEA**	MDA-MB-231	n.d.	No growth inhibition <10 µM	CB1-R	[53]
	MCF-7 EFM-19 BT-474 T-47D	0.5 µM 1.5 ± 0.3 µM 1.9 µM 1.9 µM	Cell cycle arrest, inhibition of G1/S transition	CB1-R	[54]
	MCF-7 EFM-19	1.4 ± 0.9 µM 1.5 ± 0.3 µM	Inhibition of adenylyl cyclase and activation of MAPK, thereby exerting a downregulation of PRLr and trk	n.d.	[55]
	MCF-7 T-47D	1.4 ± 0.9 µM 1.9 ± 0.2 µM	Inhibition of proliferation, inhibition of forskolin-induced cAMP formation, stimulation of RAF1 translocation and MAPK activity	CB1-R CB2-R TRPV	[56]
	MDA-MB-231	n.d.	Regulation of lipid rafts	CB1-R	[57]
**2-AG**	EFM-19	n.d.	Cell cycle arrest, inhibition of G1/S transition	CB1-R	[54]
	MCF-7 T-47D	1.4 ± 0.3 µM 5.0 ± 1.1 µM	Inhibition of proliferation, inhibition of forskolin-induced cAMP formation, stimulation of RAF1 translocation and MAPK activity	CB1-R CB2-R TRPV	[56]
**Met-F-AEA**	MDA-MB-231 T-47D	n.d.	Inhibition of adhesion and migration on type IV collagen without modifying integrin expression	CB1-R	[53]
	MDA-MB-231	n.d.	Inhibition of proliferation by degradation of b-catenin and decrease in cyclin D1, c-Myc and MMP-2 Cell cycle arrest, inhibition of G1/S transition Upregulation of E-cadherin accompanied by the reduction of vimentin, fibronectin and N-cadherin	CB1-R	[58]
	MDA-MB-231	n.d.	Inhibition of angiogenesis by the reduction of pro-angiogenic factors VEGF Reduction of metalloproteinases, TIMP1 and TIMP2	n.d.	[59]
**(R)-Met-AEA**	EFM-19	0.8 µM	Cell cycle arrest, inhibition of G1/S transition	CB1-R	[54]

2-AG: 2-Arachidonoylglycerol; AEA: anandamide; Akt: protein kinase B; AMP: adenosine monophosphate; CBC: cannabichromene; CBD: cannabidiol; CBDA: cannabidiolic acid; CBN: cannabinol; CBG: cannabigerol; CB-R: cannabinoid receptor; Cdc: cell division control; COX: cyclooxygenase; GTP: guanosine triphosphate; IC: inhibitory concentration; ID-1: inhibitor of DNA binding 1; IFN: interferon; IL: interleukin; MAPK: mitogen-activated protein kinase; Met-F-AEA: 2-methyl-2’-F-anandamide; MMP: matrix metalloproteinase; mTOR: mammalian target of rapamycin; Myc: avian virus myelocytomatosis; n.d.: not determined; n.m.: non-malignant; PPAR: peroxisome proliferator-activated receptor; PRLr: prolactin receptor; RAF: proto-oncogene serine/threonine-protein kinase; Rho: transforming protein RhoA; SHARP: SMART/HDAC1 associated repressor protein; TGF: tumor growth factor; Th: T helper; THC: tetrahydrocannabinol; THCA: tetrahydrocannabinolic acid; TIMP: tissue inhibitor of metalloproteinases; trk: tyrosin kinase; TRPV: transient receptor potential cation channels; VEGF: vascular endothelial growth factor.

## Abbreviations

2-AG	2-Arachidonoylglycerol
AA	Arachidonic acid
AEA	N-arachindonoylethanolamine (Anandamide)
AIDS	Acquired Immune Deficiency Syndrome
Akt	Protein kinase B
cAMP	cyclic adenosine monophosphate
CB	Cannabinoid
CBN	Cannabinol
CB1-R	Cannabinoid receptor-1
CB2-R	Cannabinoid receptor-2
CBD	Cannibidiol
CBDA	Cannabidiolic acid
CB-R	Cannabinoid receptor
Cdc	Cell division control
COX	Cyclooxygenase
DAG	Diacylglycerol
E2	17β-Estradiol
EGF	epidermal growth factor
EGFR	epidermal growth factor receptor
ER	Estrogen receptor
*erb*-B2	Epidermal growth factor receptor
ERK	Extracellular-signal-regulated kinase
FAAH	Fatty acid amide hydrolase
GPR	G-protein coupled receptor
GPER	G-protein coupled for estrogen
HER2	human epidermal growth factor receptor 2
ID-1	inhibitor of DNA binding 1
IL	Interleukin
MAGL	Monoacylglycerol lipase
MAPK	Mitogen-activated protein kinase
MMP	Metalloproteinases
mTOR	Mammalian target of rapamycin
NAPE	N-acyl-phosphatidylethanolamine
NAT	N-acyltransferase
NGF	Nerve growth factor
PI3K	Phosphoinoside 3-kinase
PLC	Phospholipase C
PPAR	Peroxisome proliferator-activated receptor
Phyto-CBs	Phytocannabinoids
ROS	Reactive oxygen species
SERM	Selective estrogen receptor modulator
SRC	Proto-oncogene tyrosine-protein kinase Src
STAT	Signal transducer and activator of transcription
THC	Delta-9-Tetrahydrocannabinol
THCA	Delta-9-tetrahydrocannabinolic acid
TNF	Tumor necrosis factor
VEGF	Vascular endothelial growth factor receptor

## References

[1] Abrams D.I. (2016). Integrating cannabis into clinical cancer care. Curr. Oncol..

[2] Bonini S.A., Premoli M., Tambaro S., Kumar A., Maccarinelli G., Memo M., Mastinu A. (2018). *Cannabis sativa*: A comprehensive ethnopharmacological review of a medicinal plant with a long history. J. Ethnopharmacol..

[3] Hanus L.O., Meyer S.M., Munoz E., Taglialatela-Scafati O., Appendino G. (2016). Phytocannabinoids: A unified critical inventory. Nat. Prod. Rep..

[4] Nuutinen T. (2018). Medicinal properties of terpenes found in *Cannabis sativa* and Humulus lupulus. Eur. J. Med. Chem..

[5] Fasinu P.S., Phillips S., ElSohly M.A., Walker L.A. (2016). Current Status and Prospects for Cannabidiol Preparations as New Therapeutic Agents. Pharmacotherapy.

[6] Jikomes N., Zoorob M. (2018). The Cannabinoid Content of Legal Cannabis in Washington State Varies Systematically Across Testing Facilities and Popular Consumer Products. Sci. Rep..

[7] Chakravarti B., Ravi J., Ganju R.K. (2014). Cannabinoids as therapeutic agents in cancer: Current status and future implications. Oncotarget.

[8] Velasco G., Sánchez C., Guzmán M. (2016). Anticancer mechanisms of cannabinoids. Curr. Oncol..

[9] Zou S., Kumar U. (2018). Cannabinoid Receptors and the Endocannabinoid System: Signaling and Function in the Central Nervous System. Int. J. Mol. Sci..

[10] Pertwee R.G. (2012). Targeting the endocannabinoid system with cannabinoid receptor agonists: Pharmacological strategies and therapeutic possibilities. Philos. Trans. R. Soc. Lond. B Biol. Sci..

[11] Di Marzo V., De Petrocellis L., Bisogno T. (2005). The biosynthesis, fate and pharmacological properties of endocannabinoids. Handbook of Experimental Pharmacology.

[12] De Petrocellis L., Cascio M.G., Di Marzo V. (2004). The endocannabinoid system: A general view and latest additions. Br. J. Pharmacol..

[13] Solinas M., Goldberg S.R., Piomelli D. (2008). The endocannabinoid system in brain reward processes. Br. J. Pharmacol..

[14] Mallet C., Dubray C., Duale C. (2016). FAAH inhibitors in the limelight, but regrettably. Int. J. Clin. Pharm..

[15] Salort G., Alvaro-Bartolome M., Garcia-Sevilla J.A. (2017). Regulation of cannabinoid CB2 receptor constitutive activity in vivo: Repeated treatments with inverse agonists reverse the acute activation of JNK and associated apoptotic signaling in mouse brain. Psychopharmacology.

[16] Preet A., Qamri Z., Nasser M.W., Prasad A., Shilo K., Zou X., Groopman J.E., Ganju R.K. (2011). Cannabinoid receptors, CB1 and CB2, as novel targets for inhibition of non-small cell lung cancer growth and metastasis. Cancer Prev. Res..

[17] Howlett A.C. (2005). Cannabinoid receptor signaling. Handbook of Experimental Pharmacology.

[18] Galve-Roperh I., Sanchez C., Cortes M.L., Gomez del Pulgar T., Izquierdo M., Guzman M. (2000). Anti-tumoral action of cannabinoids: Involvement of sustained ceramide accumulation and extracellular signal-regulated kinase activation. Nat. Med..

[19] Dalton G.D., Howlett A.C. (2012). Cannabinoid CB1 receptors transactivate multiple receptor tyrosine kinases and regulate serine/threonine kinases to activate ERK in neuronal cells. Br. J. Pharmacol..

[20] Leyva-Illades D., Demorrow S. (2013). Orphan G protein receptor GPR55 as an emerging target in cancer therapy and management. Cancer Manag. Res..

[21] Pertwee R.G., Howlett A.C., Abood M.E., Alexander S.P., Di Marzo V., Elphick M.R., Greasley P.J., Hansen H.S., Kunos G., Mackie K. (2010). International Union of Basic and Clinical Pharmacology. LXXIX. Cannabinoid receptors and their ligands: Beyond CB(1) and CB(2). Pharm. Rev..

[22] Izzo A.A., Borrelli F., Capasso R., Di Marzo V., Mechoulam R. (2009). Non-psychotropic plant cannabinoids: New therapeutic opportunities from an ancient herb. Trends Pharmacol. Sci..

[23] Casajuana Koguel C., Lopez-Pelayo H., Balcells-Olivero M.M., Colom J., Gual A. (2018). Psychoactive constituents of cannabis and their clinical implications: A systematic review. Adicciones.

[24] Pertwee R.G. (2008). The diverse CB1 and CB2 receptor pharmacology of three plant cannabinoids: delta9-tetrahydrocannabinol, cannabidiol and delta9-tetrahydrocannabivarin. Br. J. Pharmacol..

[25] Huestis M.A. (2007). Human cannabinoid pharmacokinetics. Chem. Biodivers..

[26] McPartland J.M. (2001). Cannabis and Eicosanoids: A Review of Molecular Pharmacology. J. Cannabis Ther..

[27] Shrivastava A., Kuzontkoski P.M., Groopman J.E., Prasad A. (2011). Cannabidiol induces programmed cell death in breast cancer cells by coordinating the cross-talk between apoptosis and autophagy. Mol. Cancer.

[28] Hegde V.L., Singh U.P., Nagarkatti P.S., Nagarkatti M. (2015). Critical Role of Mast Cells and Peroxisome Proliferator-Activated Receptor gamma in the Induction of Myeloid-Derived Suppressor Cells by Marijuana Cannabidiol In Vivo. J. Immunol..

[29] Kogan N.M. (2005). Cannabinoids and cancer. Mini Rev. Med. Chem..

[30] Lu Y., Anderson H.D. (2017). Cannabinoid signaling in health and disease. Can. J. Physiol. Pharmacol..

[31] Solinas M., Massi P., Cantelmo A.R., Cattaneo M.G., Cammarota R., Bartolini D., Cinquina V., Valenti M., Vicentini L.M., Noonan D.M. (2012). Cannabidiol inhibits angiogenesis by multiple mechanisms. Br. J. Pharmacol..

[32] Pellati F., Borgonetti V., Brighenti V., Biagi M., Benvenuti S., Corsi L. (2018). *Cannabis sativa* L. and Nonpsychoactive Cannabinoids: Their Chemistry and Role against Oxidative Stress, Inflammation, and Cancer. BioMed Res. Int..

[33] Nahtigal I., Blake A., Hand A., Florentinus-Mefailoski A., Hashemi H., Friedberg J. (2016). The Pharmacological Properties of Cannabis.

[34] Borrelli F., Fasolino I., Romano B., Capasso R., Maiello F., Coppola D., Orlando P., Battista G., Pagano E., Di Marzo V. (2013). Beneficial effect of the non-psychotropic plant cannabinoid cannabigerol on experimental inflammatory bowel disease. Biochem. Pharmacol..

[35] Bab I., Zimmer A., Melamed E. (2009). Cannabinoids and the skeleton: From marijuana to reversal of bone loss. Ann. Med..

[36] Morales P., Hurst D.P., Reggio P.H. (2017). Molecular Targets of the Phytocannabinoids: A Complex Picture. Prog. Chem. Org. Nat. Prod..

[37] Castaneto M.S., Gorelick D.A., Desrosiers N.A., Hartman R.L., Pirard S., Huestis M.A. (2014). Synthetic cannabinoids: Epidemiology, pharmacodynamics, and clinical implications. Drug Alcohol Depend..

[38] Turgeman I., Bar-Sela G. (2019). Cannabis for cancer—Illusion or the tip of an eceberg: A review of the evidence for the use of Cannabis and synthetic cannabinoids in oncology. Expert Opin. Investig. Drugs.

[39] Fraguas-Sanchez A.I., Fernandez-Carballido A., Torres-Suarez A.I. (2016). Phyto-, endo- and synthetic cannabinoids: Promising chemotherapeutic agents in the treatment of breast and prostate carcinomas. Expert Opin. Investig. Drugs.

[40] Pokrywka M., Goralska J., Solnica B. (2016). Cannabinoids—A new weapon against cancer?. Postepy Higieny i Medycyny Doswiadczalnej (Online).

[41] Ramer R., Hinz B. (2017). Cannabinoids as Anticancer Drugs. Adv. Pharmacol..

[42] Bray F., Ferlay J., Soerjomataram I., Siegel R.L., Torre L.A., Jemal A. (2018). Global cancer statistics 2018: GLOBOCAN estimates of incidence and mortality worldwide for 36 cancers in 185 countries. CA Cancer J. Clin..

[43] Prat A., Pineda E., Adamo B., Galvan P., Fernandez A., Gaba L., Diez M., Viladot M., Arance A., Munoz M. (2015). Clinical implications of the intrinsic molecular subtypes of breast cancer. Breast.

[44] Caffarel M.M., Sarrió D., Palacios J., Guzmán M., Sánchez C. (2006). Δ9-Tetrahydrocannabinol Inhibits Cell Cycle Progression in Human Breast Cancer Cells through Cdc2 Regulation. Cancer Res..

[45] McKallip R.J., Nagarkatti M., Nagarkatti P.S. (2005). Delta-9-tetrahydrocannabinol enhances breast cancer growth and metastasis by suppression of the antitumor immune response. J. Immunol..

[46] McAllister S.D., Christian R.T., Horowitz M.P., Garcia A., Desprez P.Y. (2007). Cannabidiol as a novel inhibitor of Id-1 gene expression in aggressive breast cancer cells. Mol. Cancer.

[47] Ligresti A., Moriello A.S., Starowicz K., Matias I., Pisanti S., De Petrocellis L., Laezza C., Portella G., Bifulco M., Di Marzo V. (2006). Antitumor activity of plant cannabinoids with emphasis on the effect of cannabidiol on human breast carcinoma. J. Pharm. Exp..

[48] Sultan A.S., Marie M.A., Sheweita S.A. (2018). Novel mechanism of cannabidiol-induced apoptosis in breast cancer cell lines. Breast.

[49] Takeda S. (2013). Medicinal chemistry and pharmacology focused on cannabidiol, a major component of the fiber-type cannabis. Yakugaku Zasshi.

[50] Takeda S., Misawa K., Yamamoto I., Watanabe K. (2008). Cannabidiolic acid as a selective cyclooxygenase-2 inhibitory component in cannabis. Drug Metab. Dispos..

[51] Takeda S., Okajima S., Miyoshi H., Yoshida K., Okamoto Y., Okada T., Amamoto T., Watanabe K., Omiecinski C.J., Aramaki H. (2012). Cannabidiolic acid, a major cannabinoid in fiber-type cannabis, is an inhibitor of MDA-MB-231 breast cancer cell migration. Toxicol. Lett..

[52] Takeda S., Okazaki H., Ikeda E., Abe S., Yoshioka Y., Watanabe K., Aramaki H. (2014). Down-regulation of cyclooxygenase-2 (COX-2) by cannabidiolic acid in human breast cancer cells. J. Toxicol. Sci..

[53] Grimaldi C., Pisanti S., Laezza C., Malfitano A.M., Santoro A., Vitale M., Caruso M.G., Notarnicola M., Iacuzzo I., Portella G. (2006). Anandamide inhibits adhesion and migration of breast cancer cells. Exp. Cell Res..

[54] De Petrocellis L., Melck D., Palmisano A., Bisogno T., Laezza C., Bifulco M., Di Marzo V. (1998). The endogenous cannabinoid anandamide inhibits human breast cancer cell proliferation. Proc. Natl. Acad. Sci. USA.

[55] Melck D., Rueda D., Galve-Roperh I., De Petrocellis L., Guzman M., Di Marzo V. (1999). Involvement of the cAMP/protein kinase A pathway and of mitogen-activated protein kinase in the anti-proliferative effects of anandamide in human breast cancer cells. FEBS Lett..

[56] Melck D., De Petrocellis L., Orlando P., Bisogno T., Laezza C., Bifulco M., Di Marzo V. (2000). Suppression of nerve growth factor Trk receptors and prolactin receptors by endocannabinoids leads to inhibition of human breast and prostate cancer cell proliferation. Endocrinology.

[57] Sarnataro D., Grimaldi C., Pisanti S., Gazzerro P., Laezza C., Zurzolo C., Bifulco M. (2005). Plasma membrane and lysosomal localization of CB1 cannabinoid receptor are dependent on lipid rafts and regulated by anandamide in human breast cancer cells. FEBS Lett..

[58] Laezza C., D’Alessandro A., Paladino S., Maria Malfitano A., Chiara Proto M., Gazzerro P., Pisanti S., Santoro A., Ciaglia E., Bifulco M. (2012). Anandamide inhibits the Wnt/beta-catenin signalling pathway in human breast cancer MDA MB 231 cells. Eur. J. Cancer.

[59] Picardi P., Ciaglia E., Proto M., Pisanti S. (2014). Anandamide inhibits breast tumor-induced angiogenesis. Transl. Med. UniSa.

[60] Qamri Z., Preet A., Nasser M.W., Bass C.E., Leone G., Barsky S.H., Ganju R.K. (2009). Synthetic cannabinoid receptor agonists inhibit tumor growth and metastasis of breast cancer. Mol. Cancer.

[61] Caffarel M.M., Andradas C., Mira E., Perez-Gomez E., Cerutti C., Moreno-Bueno G., Flores J.M., Garcia-Real I., Palacios J., Manes S. (2010). Cannabinoids reduce ErbB2-driven breast cancer progression through Akt inhibition. Mol. Cancer.

[62] Perez-Gomez E., Andradas C., Blasco-Benito S., Caffarel M.M., Garcia-Taboada E., Villa-Morales M., Moreno E., Hamann S., Martin-Villar E., Flores J.M. (2015). Role of cannabinoid receptor CB2 in HER2 pro-oncogenic signaling in breast cancer. J. Natl. Cancer Inst..

[63] Ursini-Siegel J., Schade B., Cardiff R.D., Muller W.J. (2007). Insights from transgenic mouse models of ERBB2-induced breast cancer. Nat. Rev. Cancer.

[64] Ford L.A., Roelofs A.J., Anavi-Goffer S., Mowat L., Simpson D.G., Irving A.J., Rogers M.J., Rajnicek A.M., Ross R.A. (2010). A role for L-alpha-lysophosphatidylinositol and GPR55 in the modulation of migration, orientation and polarization of human breast cancer cells. Br. J. Pharmacol..

[65] Zhang J., Zhang S., Liu Y., Su M., Ling X., Liu F., Ge Y., Bai M. (2018). Combined CB2 Receptor Agonist and Photodynamic Therapy Synergistically Inhibit Tumor Growth in Triple Negative Breast Cancer. Photodiagn. Photodyn..

[66] Wu H.Y., Huang C.H., Lin Y.H., Wang C.C., Jan T.R. (2018). Cannabidiol induced apoptosis in human monocytes through mitochondrial permeability transition pore-mediated ROS production. Free Radic. Biol. Med..

[67] Bouquie R., Deslandes G., Mazare H., Cogne M., Mahe J., Gregoire M., Jolliet P. (2018). Cannabis and anticancer drugs: Societal usage and expected pharmacological interactions—A review. Fundam. Clin. Pharm..

[68] Murase R., Kawamura R., Singer E., Pakdel A., Sarma P., Judkins J., Elwakeel E., Dayal S., Martinez-Martinez E., Amere M. (2014). Targeting multiple cannabinoid anti-tumour pathways with a resorcinol derivative leads to inhibition of advanced stages of breast cancer. Br. J. Pharmacol..

[69] Scott K.A., Dalgleish A.G., Liu W.M. (2017). Anticancer effects of phytocannabinoids used with chemotherapy in leukaemia cells can be improved by altering the sequence of their administration. Int. J. Oncol..

[70] Yasmin-Karim S., Moreau M., Mueller R., Sinha N., Dabney R., Herman A., Ngwa W. (2018). Enhancing the Therapeutic Efficacy of Cancer Treatment With Cannabinoids. Front. Oncol..

[71] Radin D.P., Patel P. (2016). Delineating the molecular mechanisms of tamoxifen’s oncolytic actions in estrogen receptor-negative cancers. Eur. J. Pharmacol..

[72] Hart C.D., Migliaccio I., Malorni L., Guarducci C., Biganzoli L., Di Leo A. (2015). Challenges in the management of advanced, ER-positive, HER2-negative breast cancer. Nat. Rev. Clin. Oncol..

[73] Elbaz M., Nasser M.W., Ravi J., Wani N.A., Ahirwar D.K., Zhao H., Oghumu S., Satoskar A.R., Shilo K., Carson W.E. (2015). Modulation of the tumor microenvironment and inhibition of EGF/EGFR pathway: Novel anti-tumor mechanisms of Cannabidiol in breast cancer. Mol. Oncol..

[74] Blasco-Benito S., Seijo-Vila M., Caro-Villalobos M., Tundidor I., Andradas C., Garcia-Taboada E., Wade J., Smith S., Guzman M., Perez-Gomez E. (2018). Appraising the “entourage effect”: Antitumor action of a pure cannabinoid versus a botanical drug preparation in preclinical models of breast cancer. Biochem. Pharmacol..

[75] Mohammadpour F., Ostad S.N., Aliebrahimi S., Daman Z. (2017). Anti-invasion Effects of Cannabinoids Agonist and Antagonist on Human Breast Cancer Stem Cells. Iran. J. Pharm. Res..

[76] Pan H., Mukhopadhyay P., Rajesh M., Patel V., Mukhopadhyay B., Gao B., Haskó G., Pacher P. (2009). Cannabidiol attenuates cisplatin-induced nephrotoxicity by decreasing oxidative/nitrosative stress, inflammation, and cell death. J. Pharmacol. Exp. Ther..

[77] Baselga J., Swain S.M. (2009). Novel anticancer targets: Revisiting ERBB2 and discovering ERBB3. Nat. Rev. Cancer.

[78] Portella G., Laezza C., Laccetti P., De Petrocellis L., Di Marzo V., Bifulco M. (2003). Inhibitory effects of cannabinoid CB1 receptor stimulation on tumor growth and metastatic spreading: Actions on signals involved in angiogenesis and metastasis. FASEB J..

[79] Chen S.-H., Cheung C.H.A. (2018). Challenges in Treating Estrogen Receptor-Positive Breast Cancer. IntechOpen.

[80] Kogan N.M., Schlesinger M., Priel E., Rabinowitz R., Berenshtein E., Chevion M., Mechoulam R. (2007). HU-331, a novel cannabinoid-based anticancer topoisomerase II inhibitor. Mol. Cancer.

[81] Prossnitz E.R., Barton M. (2014). Estrogen biology: New insights into GPER function and clinical opportunities. Mol. Cell. Endocrinol..

[82] Hsu L.H., Chu N.M., Lin Y.F., Kao S.H. (2019). G-Protein Coupled Estrogen Receptor in Breast Cancer. Int. J. Mol. Sci..

[83] Dobovisek L., Hojnik M., Ferk P. (2016). Overlapping molecular pathways between cannabinoid receptors type 1 and 2 and estrogens/androgens on the periphery and their involvement in the pathogenesis of common diseases (Review). Int. J. Mol. Med..

[84] Sharma D., Kumar S., Narasimhan B. (2018). Estrogen alpha receptor antagonists for the treatment of breast cancer: A review. Chem. Cent. J..

[85] Wang M., Chen H., Wu K., Ding A., Zhang M., Zhang P. (2018). Evaluation of the prognostic stage in the 8th edition of the American Joint Committee on Cancer in locally advanced breast cancer: An analysis based on SEER 18 database. Breast.

[86] Tan W., Li Q., Chen K., Su F., Song E., Gong C. (2016). Estrogen receptor beta as a prognostic factor in breast cancer patients: A systematic review and meta-analysis. Oncotarget.

[87] Ruh M.F., Taylor J.A., Howlett A.C., Welshons W.V. (1997). Failure of cannabinoid compounds to stimulate estrogen receptors. Biochem. Pharmacol..

[88] Takeda S., Yoshida K., Nishimura H., Harada M., Okajima S., Miyoshi H., Okamoto Y., Amamoto T., Watanabe K., Omiecinski C.J. (2013). Delta(9)-Tetrahydrocannabinol disrupts estrogen-signaling through up-regulation of estrogen receptor beta (ERbeta). Chem. Res. Toxicol..

[89] Mungenast F., Thalhammer T. (2014). Estrogen biosynthesis and action in ovarian cancer. Front. Endocrinol..

[90] Prather P.L., FrancisDevaraj F., Dates C.R., Greer A.K., Bratton S.M., Ford B.M., Franks L.N., Radominska-Pandya A. (2013). CB1 and CB2 receptors are novel molecular targets for Tamoxifen and 4OH-Tamoxifen. Biochem. Biophys. Res. Commun..

[91] Franks L.N., Ford B.M., Fujiwara T., Zhao H., Prather P.L. (2018). The tamoxifen derivative ridaifen-B is a high affinity selective CB2 receptor inverse agonist exhibiting anti-inflammatory and anti-osteoclastogenic effects. Toxicol. Appl. Pharmacol..

[92] Kumar P., Song Z.H. (2014). CB2 cannabinoid receptor is a novel target for third-generation selective estrogen receptor modulators bazedoxifene and lasofoxifene. Biochem. Biophys. Res. Commun..

[93] Franks L.N., Ford B.M., Prather P.L. (2016). Selective Estrogen Receptor Modulators: Cannabinoid Receptor Inverse Agonists with Differential CB1 and CB2 Selectivity. Front. Pharmacol..

[94] Ma H., Yan D., Wang Y., Shi W., Liu T., Zhao C., Huo S., Duan J., Tao J., Zhai M. (2019). Bazedoxifene exhibits growth suppressive activity by Targeting IL-6/GP130/STAT3 Signaling in Hepatocellular Carcinoma. Cancer Sci..

[95] Cyr C., Arboleda M.F., Aggarwal S.K., Balneaves L.G., Daeninck P., Neron A., Prosk E., Vigano A. (2018). Cannabis in palliative care: Current challenges and practical recommendations. Ann. Palliat. Med..

[96] Davis M.P. (2016). Cannabinoids for Symptom Management and Cancer Therapy: The Evidence. J. Natl. Compr. Cancer Netw..

